# Child injuries in Ethiopia: A review of the current situation with projections

**DOI:** 10.1371/journal.pone.0194692

**Published:** 2018-03-27

**Authors:** Qingfeng Li, Olakunle Alonge, Collene Lawhorn, Yirga Ambaw, Smita Kumar, Troy Jacobs, Adnan A. Hyder

**Affiliations:** 1 Johns Hopkins International Injury Research Unit, Department of International Health, Johns Hopkins Bloomberg School of Public Health, Baltimore, Maryland, United States of America; 2 Bureau for Global Health, Office of Health, Infectious Diseases & Nutrition, United States Agency for International Development, Washington, DC, United States of America; 3 Office of Health, AIDS, Population and Nutrition, United States Agency for International Development, Ethiopia, Addis Ababa, Ethiopia; Karolinska Institutet, SWEDEN

## Abstract

**Background:**

Heavy burden of child injuries and lack of policy response in Ethiopia call for an improved understanding of the situation and development of action plans from multiple governmental agencies and stakeholders.

**Methods:**

A consortium of international and Ethiopian researchers and stakeholders used extensive literature review and mixed analytical methods to estimate and project the burden of fatal and non-fatal child unintentional injuries in Ethiopia from 2015 to 2030. Estimates were derived for children aged 0–14 years. Data sources include a longitudinal study conducted by the Central Statistics Agency of Ethiopia and the World Bank as well as model-based estimates from World Health Organization 2017 and Global Burden of Disease 2016 project.

**Results:**

Injuries caused about 25 thousand deaths among 0-14-year olds in Ethiopia in 2015. The leading cause of fatal child unintentional injuries in Ethiopia was road-traffic injuries, followed by fire, heat and hot substances and drowning. The death rate due to injuries among 0–14 years olds was about 50 percent higher in males than females. Rural children were exposed to a greater risk of injury than their urban peers. The longitudinal survey suggests that the incidence rate of child injuries increased during the period 2011–2014. The annual mortality caused by injuries is projected to increase from 10,697 in 2015 to 11,279 in 2020 and 11,989 in 2030 among children under 5 years, an increase of 12 percentage points in 15 years. The number of deaths among 0-14-year olds will be 26,463, 27,807, and 30,364 respectively in 2015, 2020, and 2030.

**Conclusions:**

As the first multisectoral collaboration on child injuries in Ethiopia, this study identified gaps in understanding of the burden of child injuries in Ethiopia. In consultation with Ethiopian government and other stakeholders, we propose starting an injury surveillance system at health clinics and hospitals and building an intervention package based on existing platforms.

## Introduction

In many low-and middle-income countries (LMICs), children face a greater risk of death and disability from injuries than their peers in the high-income world. According to the World Health Organization (WHO) global health estimates (released in 2017), injuries caused 4.92 million deaths in 2015, and of those 366 thousand were children under 5 years, and 352 thousand were children aged 5–14 years [1]. The years of life lost (YLL) due to injuries was 249 million each year globally. These high injury death rates and YLL are a tremendous health, economic, and social burden globally [2]. However, injury prevention has received less global attention from government agencies, donors and nongovernmental organizations (NGO) compared to other public health issues.

In Ethiopia, the 2015 age-standardized mortality rate for injuries was 84 per 100,000 population; and injuries accounted for 4,981 YLL per 100,000 population [2]. These rates were higher than the global average, implying that Ethiopia is one of the countries driving the high global burden of injuries. A large proportion of injury deaths in Ethiopia occur among children. It is estimated that of the 83,516 total injury deaths in 2015, 13,550 deaths were among children under 5 years and 11,684 among children 5–14 years old.^1^ It is therefore important for stakeholders to pay attention and take action to address the burden of injuries among children in Ethiopia.

The burden of injuries among children in Ethiopia described so far is based on model-based data from the WHO, and omits key information on the risk factors of child injuries from the population. Hence, there are still significant gaps in understanding the true burden of child injuries in Ethiopia. To date, there has been no comprehensive review of literature and synthesis of evidence that would help elucidate some of the major risk factors for child injuries in Ethiopia. In this paper, we provide a more robust estimate of the burden of child injuries and associated trends in Ethiopia through a review and triangulation of all available data sources (including recent model-based and real data) for Ethiopia. We also describe a synthesis of the major risk factors of child injuries for Ethiopia and compare that to global average based on review of relevant literature.

## Data and methods

### Data and literature review

An extensive search was conducted for data, studies and government reports on injuries among children 0–14 years of age in Ethiopia. For the data search, we consulted with various relevant ministries and agencies conducting health research and primary data collection in Ethiopia, including the Ethiopian Central Statistical Agency (CSA). Additionally, we searched for accessible web-based data sources through Google. For the literature review, we described keywords of interest including Ethiopia and concepts of injury mechanism such as road traffic crashes, poisoning, falls, fire, heat and hot substance, drowning, exposure to forces of nature, and violence. The keywords and their possible combinations were used to search articles published between 2000 and 2017 in PUBMED, EMBASE, POPLINE, CINAHL, Scopus, and Google Scholar databases.

In each identified data source, we counted all fatal unintentional injuries and only non-fatal unintentional injuries that required medical treatment or the injured individual reported loss of days from regular activities. Because medical services are not readily accessible to all households and children in Ethiopia, we also counted the injuries that required medical treatment, but were not treated due to accessibility and affordability issues. Minor injuries without serious consequences were excluded. We aggregated data derived from standard indicators that are commonly used to measure the burden of injuries, including disability-adjusted life-year (DALY), years lost due to disability (YLD), and YLL.

#### Global model estimates

We identified two major sources of aggregate data on child injuries in Ethiopia: The Global Burden of Diseases (GBD) 2016 and Global Health Estimates (GHE) 2017. The GBD and GHE used different methodologies and data sources, and therefore yielded different national, regional and global estimates. We compared these two data sources in an attempt to provide more robust conclusions on the count of child injury mortality and morbidity in Ethiopia. In order to calculate mortality and morbidity rates, we needed population exposure data; this was obtained from the United Nations’ World Population Prospects (WPP) data for 2017 [3]. A detailed description of methodologies and inputs of the GBD, GHE and WPP data sources is beyond the scope of this report. However, these data sources are developed and regularly updated by internationally renowned organizations and the estimates have been extensively used by policy makers and academic researchers around the world. The present study focuses on children 0–14 years old.

#### National surveys

In addition to GBD and GHE model-based data, we estimated injury rates for children 0–14 years from a longitudinal survey in Ethiopia. The survey included two-waves of data collection and was jointly conducted by the Central Statistics Agency of Ethiopia (CSA) and the World Bank. The first wave, called Ethiopian Rural Socioeconomic Survey (ERSS), was conducted between September 2011 and March 2012. It was based on a sample representative of all households in rural areas and small towns. The second wave, called the Ethiopia Socioeconomic Survey (ESS), was conducted between September 2013 and April 2014. The sample of ESS was representative of the whole country, including rural areas, small towns, and large towns. The ESS collected information only on non-fatal injuries. To our knowledge, the ESS is the only survey that collected information on child injuries from a nationally representative sample in Ethiopia. The analysis in this study is based on two of the injury-related questions asked in the surveys: *during the past 4 weeks*, *has [name] suffered from an illness or injury*? *If yes*, *what was the sickness/injury [name] faced*? We converted the incidence count from a 4-week basis to an annual basis by simply multiplying the observed count by a factor of 52 divided by 4. This conversion approach is commonly applied in the literature to generate annualized rates and minimize bias that may result from estimates based on longer recall periods [4, 5].

### Mixed-methods projection

Based on the survey-based estimates, we projected the burden under a hypothetical scenario where the incidence rate of child unintentional injuries and fatalities remain at the same level as in ESS 2013–2014. Since ESS surveys only collected morbidity data, we estimated injury mortality rates based on ratio of non-fatal to fatal unintentional injuries from literature. A study from the United States estimated that there was one fatality for every 45 children that required hospitalization due to injuries and 1300 that were seen in an emergency department among children under 19 years of age [6, 7]. Another study conducted in 5 LMICs in Asia on over 500,000 thousand households and 2.5 million people estimated that there was one fatality for every 34 children who sought medical care or missed school or work due to injuries among children 18 years and under [8]. Unfortunately, no such study is available for Ethiopia in the literature. Hence, the ratio used to estimate the number of deaths due to injuries in this paper was based on the study from other LMICs, i.e. 1 injury death to 34 injury cases.

All analyses were done in Stata 15 SE.

## Results

### Model-based estimates

Due to the differences in model assumptions and data inputs, GBD 2016 and GHE 2017 provided varying estimates. The GHE 2017 estimated that 13,550 children under five years of age and an additional 11,687 children aged 5–14 years died from injuries in 2015 (Table 1). The estimates from GBD 2016 are less than half of the GHE results at 4,922 deaths for 0–4 years old and 4,354 deaths for 5–14 years old.

**Table 1:** Number and rate of mortality due to injuries by age group in Ethiopia and the world from Global Burden of Disease (GBD) 2016, Global Health Estimates (GHE) 2017, and Ethiopian Socioeconomic Survey (ESS) 2013–2014.

Number/Rate	Ethiopia			World	
	GBD	GHE	ESS	GBD	GHE
Reference year	2015	2015	2013–2014	2015	2015
Number of deaths					
0–4 years	4,922	13,550	10,502	262,792	206,861
5–14 years	4,354	11,687	15,600	221,593	218,060
**0–14 years**	9,276	25,237	25,519	484,385	424,921
Death rate (per 100,000)					
0–4 years	33	93	74	41	60
5–14 years	16	44	60	18	34
**0–14 years**	22	61	64	26	43
Number of injuries					
0–4 years	n/a	n/a	357,071	n/a	n/a
5–14 years	n/a	n/a	530,400	n/a	n/a
**0–14 years**	n/a	n/a	867,661	n/a	n/a
Injury rate (per 100,000)					
0–4 years	n/a	n/a	2,520	n/a	n/a
5–14 years	n/a	n/a	2,040	n/a	n/a
**0–14 years**	n/a	n/a	2,160	n/a	n/a

**Fig 1:**
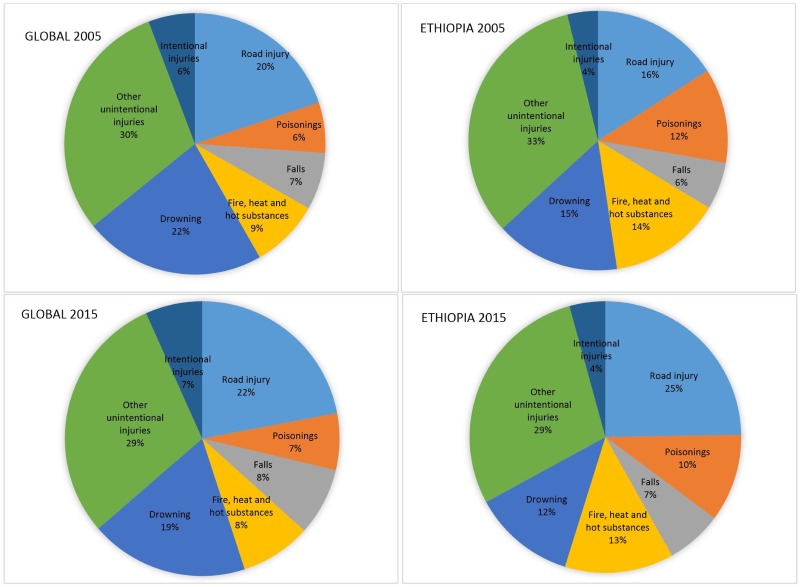
Under-five injury deaths by cause globally and in Ethiopia, 2005 and 2015. Source: Global Health Estimates 2017.

Fig 1 illustrates under-five deaths by cause, globally and in Ethiopia in 2005 and 2015, according to GHE 2017. Road traffic injuries caused about one fifth of global deaths in both years; that percentage increased for Ethiopia from 16% in 2005 to 25% in 2015, which is consistent with the rapid motorization of the country during this period. Injuries caused by fire, heat and hot substance comprised about 9% of the global injuries; this proportion is however much larger at 14% and 13% in Ethiopia in 2005 and 2015, respectively. The proportion of injuries caused by drowning is smaller in Ethiopia, and it experienced a slight decrease from 15% in 2005 to 12% in 2015. The proportionate mortality due to drowning was lower in Ethiopia than global average. It should be noted that injuries related to intentional injuries shown here are likely underestimated because of reporting issues, and that requires additional attention to get a better understanding of the prevalence.

**Fig 2:**
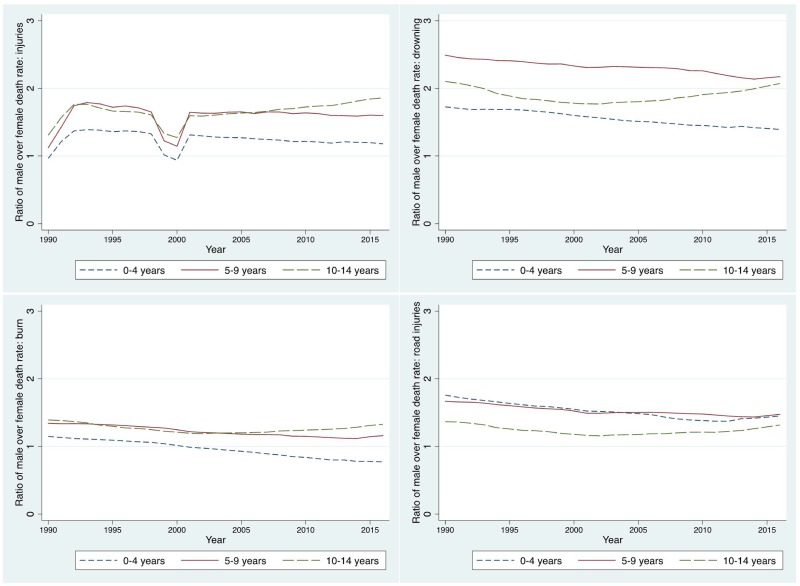
The ratio of male over female fatality rate due to all injuries (upper left), drowning (upper right), fire, heat and hot substance (lower left), and road injuries (lower right) by age group in Ethiopia: 1990–2016. Source: Global Burden of Disease 2016.

There was substantial gender difference in injury risk. The risk for all types of injury among male children is higher than among female children (Fig 2). The gender ratio comparing male to female drowning is slightly over 1.5 for children aged 0–4 and 5–9 years, and about 2 for children aged 10–14 year olds. Gender disparity is relatively smaller for injuries caused by fire, burn, and hot substance; it is below one for 10–14 years in recent years. For road injuries, the ratio fluctuated lightly around 1.5 from 1990 to 2015 among children under five years old, and has been stable at around 1.7 for 5-9-year olds. A large increase was observed for 10-14-year olds, from 1.6 to 1.9 during the period of time. There was a dip around 2000, but the ratio was still above, except for 1–14 years, which is right below one in 2000.

**Fig 3:**
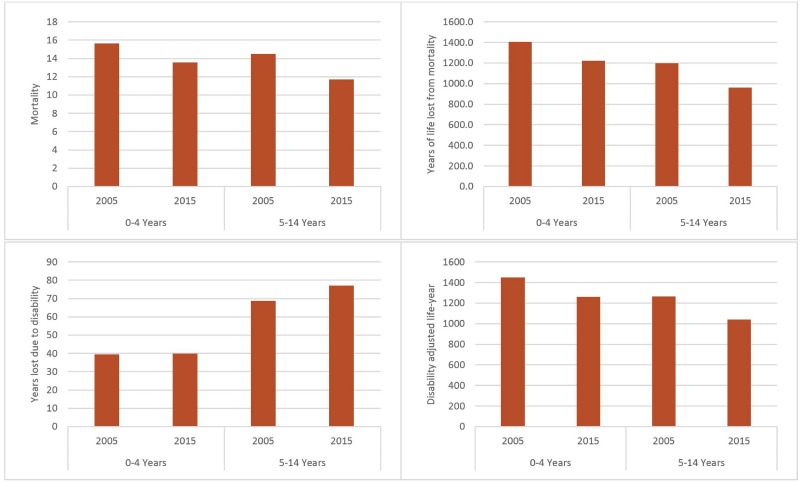
Four measures of health burden of injuries in Ethiopia (thousand), 2005 and 2015. Source: Global Health Estimates 2017.

**Fig 4:**
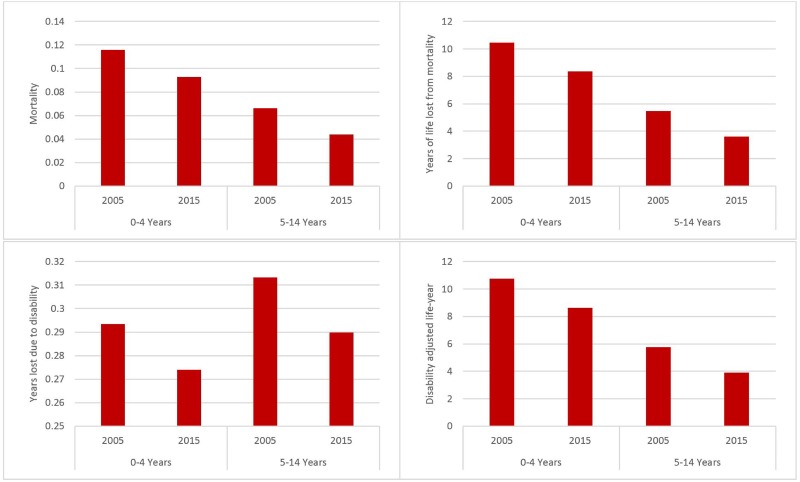
Four measures of health burden of injuries in Ethiopia (rate per 100,000): 2005 and 2015. Source: Global Health Estimates 2017.

The estimated burden of injuries from GHE 2017 are illustrated in Figs 3 and 4. The fatalities caused by injuries decreased from 15,629 in 2005 to 13,550 in 2015 for children under five years old and from 14,504 to 11,687 for children 5–14 years old. YLL and DALY were also reduced, while YLD remained steadily at 40,000 for 0–4 years old and increased from 69,000 to 77,000 for children 5–14 years old. The trend indicates that although the fatal injuries have declined, the severity of injuries might have increased over time.

Three points should be noted for the GHE estimates. First, despite the declining trend in three of the four indicators (mortality, YLL, DALY), the burden is still high as of 2015 from an international perspective. Second, morbidity increased despite the reduction in mortality burden. This may indicate that although fatal injuries decreased, the prevalence of nonfatal injuries or the severity of injuries have increased. More children may suffer lifetime health consequences of injuries sustained during childhood, which may imply long-term economic cost and loss in productivity, in addition to the health burden. The last point is that the declining trend should be interpreted with caution because model-based estimates depend on both observed data and model assumptions. Whether the change is genuine or just reflective of model assumptions needs further in-depth investigations, particularly when a declining trend is inconsistent with the findings from national surveys (reviewed below).

Because of the different modeling methodologies and data inputs, GBD 2016 provides a different picture than GHE. The burden is smaller according to GBD than GHE, which highlights the differences in their methodological approaches in deriving these estimates, but still illustrates the heavy burden in Ethiopia. The GBD estimates of mortality, YLL, and DALY are only about 40% of the corresponding GHE estimates (Table 2). GBD however estimated a higher YLD (Table 2), which may be because these two sources used different prevalence of injuries and weighting factors (severity of disability caused by injuries).

**Table 2:** Ratio of Global Burden of Disease 2016 over Global Health Estimates 2017 of four indicators of burden of injuries in Ethiopia.

Age	Year	Mortality	YLL	DALY	YLD
0–4 Years	2005	0.50	0.48	0.47	0.41
	2015	0.36	0.34	0.35	0.53
5–14 Years	2005	0.40	0.37	0.40	0.87
	2015	0.37	0.35	0.39	0.95

Notes: DALY: Disability adjusted life-year; YLL: Years of life lost due to mortality; YLD: Years lost due to disability; GBD: Global Burden of Disease; GHE: Global Health Estimate

### Survey-based estimates

The ERSS 2011–2012 and ESS 2013–2014 collected information on non-fatal unintentional injuries using identical questions. In addition, the ERSS 2011–2012 collected information on fatal unintentional injuries. Non-fatal unintentional injuries are more frequently reported than fatal injuries and may lead to long-term disability and other health issues. As a result, ignoring non-fatal injuries creates a serious underestimation of the burden of injuries.

**Fig 5:**
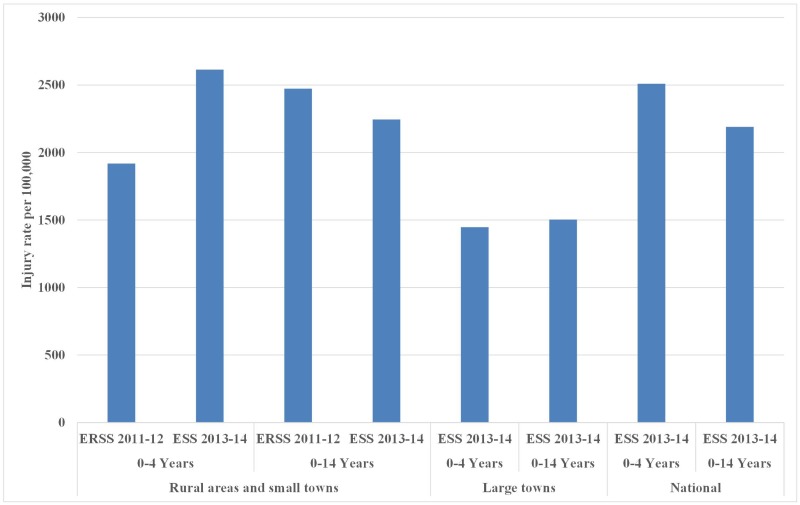
Injury rate per 100,000 children in rural areas, small towns, large towns, and the whole country: ERSS 2011–2012 and ESS 2013–2014. Source: Ethiopian Rural Socioeconomic Survey (ERSS) 2011–2012; Ethiopia Socioeconomic Survey (ESS) 2013–2014.

Fig 5 shows that between the two waves, the annual incidence rate of child injuries increased from 1,918 (95% confidence interval (CI): 860–4,270) to 2,614 (95% CI: 1,113–6,118) per 100,000 for children under-5 years in rural areas and small towns. Large towns were covered in wave 2, but not wave 1, making it infeasible to estimate the national trend.

Based on ESS data only, disparities in annual incidence rates of injuries between children living in rural, small town, and large town households were statistically significant. Incidence per 100,000 children under 5 years was higher in rural areas and small towns than in large towns (1,447, 95% CI: 156–13,219). Similar disparity was found among children aged 0–14 years, i.e. 2,245 (95%: 1,309–3,846) vs. 1,503 (95% CI: 311–7,203). Rural children carried a heavier burden of injuries than urban children.

The estimated number of deaths due to injuries obtained from survey-based results is comparable to GHE results. Based on the estimation approach noted above, we determined that 25,351 children aged 0–14 years died in Ethiopia in 2013, which is 14 percent higher than the GHE estimates. The GHE estimates also tend to underestimate the deaths caused by injuries among children 5–14 years old; the survey-based estimate of 15,472 is 67 percent higher than the corresponding GHE estimate.

The nationally representative ESS data showed that the incidence rate of injuries that required medical treatment or caused absence from regular activities is comparable among three age groups of: 0–4, 5–9, and 10–14 years. Taking into account the population size of each age group, we infer that GHE might have underestimated the deaths caused by injuries among 5-14-year olds.

### Projection of the burden of child injuries

**Fig 6:**
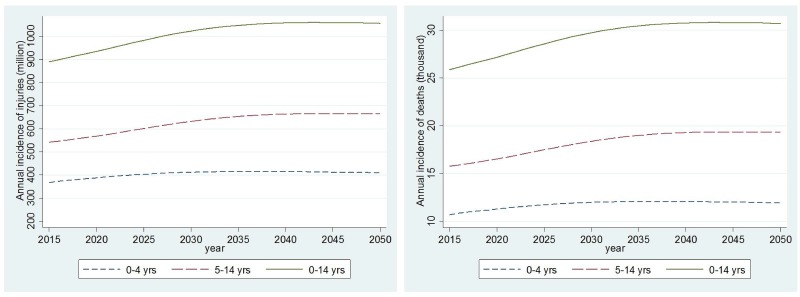
Predicted annual incidence of injuries and deaths in Ethiopia: 2015–2050.

Assuming a constant annual incidence rate, we estimated that there will be 367,963, 388,013 and 412,412 cases of injuries among children under 5 years in 2015, 2020, and 2030, respectively (Fig 6). The projected cases are 910,318, 956,547, and 1044,529 respectively in these 3 years among children aged 0–14 years. The number of fatal injuries is projected to increase from 10,697 in 2015 to 11,279 in 2020 and to 11,989 in 2030 among 0–5 year olds. The increases will be from 26,463 to 27,807 and to 30,364 among 0–14 year olds.

The sustaining and substantial increase under the hypothetical scenario of constant risk of injuries is troubling, particularly given the national and global goals of improving child survival and achieving Sustainable Development Goals by 2030.

### Risk factors for child injuries in Ethiopia

In contrast to the heavy burden illustrated in the model- and survey-based data, only a small number of risk factor studies were located in our literature review, mostly on child abuse, which is anecdotally considered the most common form of child injuries in Ethiopia [9]. Unfortunately, few studies reported on the risk factors of unintentional child injuries, which were the main focus of the burden estimate for this paper.

The prevalence of child sexual abuse was high (68.7%), among which verbal harassment filled most part (51.4%), followed by sexual intercourse (18%), and unwelcome kissing (17.1%). [10] Most of the victims are under 15 years of age and experienced psychological problems after being abused such as suicide, low degree of positive self-worth and sexual dysfunction [10–14]. Hospitals only focused on the physical healing while these psychological problems are often ignored [11]. Studies also observed a delay in reporting the assault/abuse [11]. Child marriage is another source of child sexual abuse. Study showed the consequences of child sexual abuse on psychological aspect was generally more severe in those who survived from rape or prostitution than those who were married young [15].

The incidence of child unintentional injuries in Ethiopia was found to relate to socioeconomic status, disproportionally affecting the poor [16]. Traumatic injury other than car crashes accounted for most incidences of injury (82.38%). Burns were common among children aged 5–9 years [17]. Caregiver depression and maternal depression were also linked to child injuries, particularly intentional physical injuries [18, 19].

## Discussion and conclusion

This paper is based on a consultative project involving multiple stakeholders and sectors in Ethiopia. With inputs from national and international agencies, governmental and non-governmental organizations, academic institutes and hospitals, we systematically reviewed the situation regarding the burden of child injuries in Ethiopia with projections. The consultative project is the first multi-sectoral collaboration on child injuries in Ethiopia, and one of the few in sub-Sahara Africa.

The data and studies discussed in this article illustrate that child injuries is a major public health challenge in Ethiopia, and the urgent need for action by various stakeholders including the Ethiopian national and local governments, civil society organizations, and international agencies. The consultative work upon which this paper is based represent a commendable first step to address the burden of child injuries by various stakeholders in Ethiopia. Lack of adequate data systems to yield accurate and in-depth diagnosis of the burden of child injuries is a likely contributory factor to the limited action by various stakeholders in the past.

Despite the large burden of injuries according to the model-based estimates, quality primary data on the level and trend of injuries are rarely available in Ethiopia, like in most of the developing world. Most low- and middle-income countries (LMICs) have not developed a vital registration system that enables a reliable national estimate. This is particularly the case for children aged 0–4 years. Medically certified vital registration system data were available for only 2.7% of the under-five deaths in Ethiopia in 2010 [20]. Moreover, population-level surveys on child health such as the Multiple Indicator Cluster Surveys (MICS) and the Demographic and Health Surveys (DHS), which are reliable and widely used data sources, are only starting to include a limited number of child injury questions. The sharp contrast between the heavy burden of child injuries and a serious lack of quality data to accurately assess this burden calls for more research and primary data (including both household-based surveys and hospital-based data) to better estimate rates of fatal and non-fatal injuries in Ethiopia and other similar LMICs. Improved understanding of the underlying risk factors of these injuries, as well as the disparities and inequality in the burden, is urgently needed.

Research studies that characterize the burden and contributing risk factors of child injuries are important for designing effective and targeted interventions, possibly disaggregated by rural and peri-urban/urban areas. Attention therefore needs to be paid to establishing data collection systems that enable detailed analyses and inform intervention design. Our literature review and analyses of survey data illustrated that child injuries are also a social equity issue. Data from household surveys, hospitals, and police stations indicate that children in economically disadvantaged households are more likely to be victims of injuries.

This study has limitations despite the comprehensive triangulation of various data sources and the extensive literature review. A major limitation is lack of quality data on the causes of injuries. There are several solutions to this problem. In the short term, revising that country’s Health Management Information System (HMIS) to include injury-related indicators is a cost-effective approach to rapidly collect related information. In the long term, hospital-based surveillance system is proven source for such information [21, 22]. We recommend establishing health clinic and hospital emergency and outpatient department data collection in Ethiopia. Another solution is to develop a vital registration system, starting from major cities and then gradually rolling it out throughout the country.

A particular challenge in establishing data systems is the lack of human, technical and financial capacity; which may require international support to address particularly at the early stage of implementing plans for addressing the burden of child injuries in Ethiopia. A comprehensive package with a detailed roadmap will make it possible to reduce the burden of injuries and violence in Ethiopia [23]. As a starting point, we recommend adding child injuries prevention into the training of Ethiopia’s successful Health Extension Worker Program [24]. With its extensive network of more than 38,000 health extension workers, this addition will protect children from injuries and provide experience for other injury prevention programs.

In conclusion, this triangulation of multiple data sources and prediction of future trajectory contributes to our understanding of child injuries in Ethiopia. The findings can be used to assist intervention design and policy making. Based on the study results and consultation with relevant governmental agencies in Ethiopia, we propose developing an injury surveillance system at health facilities and integrating injury prevention programs into existing platforms.
